# Gastrointestinal Bleeding in Small Intestinal Stromal Tumors: A Clinicopathological and Survival Analysis

**DOI:** 10.5152/tjg.2025.25205

**Published:** 2025-11-21

**Authors:** Fengqin Fu, Hui Pu, Weiping Huang, Lin Han, Yongbin Deng, Yadong Lai

**Affiliations:** 1 Department of Gastroenterology, Zhangzhou Municipal Hospital Affiliated to Fujian Medical University, Zhangzhou, China

**Keywords:** Gastrointestinal bleeding, gastrointestinal stromal tumors, prognosis, small intestinal stromal tumor, surgical procedures

## Abstract

**Background/Aims:**

This study analyzes the clinical pathological characteristics and prognostic factors of small intestinal stromal tumor (SIST) patients with gastrointestinal (GI) bleeding and investigates whether GI bleeding is an independent predictor of SIST prognosis.

**Materials and Methods:**

A retrospective analysis of 152 patients diagnosed with SISTs at Zhangzhou Municipal Hospital Affiliated to Fujian Medical University was conducted. Patients were divided into 2 groups based on the presence or absence of GI bleeding. Both survival and recurrence rates were calculated using univariate survival analysis and Cox regression analysis, respectively.

**Results:**

Compared with the non-GI bleeding group, the GI bleeding group showed significant differences in tumor rupture and Ki-67 abundance (*P* < .05). Survival analysis revealed that patients with GI bleeding had a shorter relapse-free survival (RFS) compared to those without GI bleeding (*P* = .016); however, GI bleeding significantly impacted RFS only in the high-risk group (*P* = .001). Cox regression analysis showed that mitotic count (*P* = .021), tumor rupture (*P* = .032), Ki67 positivity (*P* = .032), and GI bleeding (*P* = .04) were independent factors affecting the relapse-free survival rate of SISTs.

**Conclusion:**

This study shows that SIST patients with GI bleeding are more likely to experience tumor rupture and have higher Ki-67 positivity than those without GI bleeding. Mitotic count, tumor rupture, Ki-67 abundance, and GI bleeding are independent predictors of SIST prognosis. Additionally, the RFS rate for patients with GI bleeding is shorter, particularly among high-risk patients.

**Main Points**

- Patients with gastrointestinal (GI) bleeding are more likely to experience tumor rupture and have higher Ki-67 levels than those with non-GI bleeding hemorrhage small intestinal stromal tumors (SIST).
- Mitotic count, tumor rupture, Ki-67, and GI bleeding are independent prognostic factors for SIST.
- The relapse-free survival for patients with GI bleeding is shorter than that for those without, particularly in high-risk patients.

## Introduction

Gastrointestinal stromal tumors (GISTs) arise from the muscle layer of the gastrointestinal tract, mainly due to abnormal development of interstitial cells of Cajal.^1^ Gastrointestinal stromal tumor is characterized by mutations in the KIT proto-oncogene and the platelet-derived growth factor receptor alpha gene.^2^ Gastrointestinal stromal tumors were first proposed by Mazur and Clark in 1983^3^ and are the most common mesenchymal tumor of the gastrointestinal tract, accounting for 1%-3% of all gastrointestinal malignancies.^4^^,^^5^ Gastrointestinal stromal tumors can occur anywhere along the gastrointestinal tract, with the stomach being the most frequent site (58%), followed by the small intestine (28%), colorectum (10%), and esophagus (0%-6%). Rare cases may develop in the omentum and retroperitoneum.^2^^,^^6^

The small intestine is the second most common site for GISTs, known as small intestinal stromal tumors (SISTs). The malignancy degree of SISTs is much higher than that of gastric GISTs. Even with surgical treatment, recurrence and mortality rates for SISTs are still high.^7^ Therefore, assessing the recurrence risk of SISTs is crucial. According to the consensus criteria of the National Institutes of Health (NIH) and the Armed Forces Institute of Pathology, the location, size, tumor rupture status, and mitotic index of the tumor are associated with prognosis.^8^ Additionally, some studies indicate that Ki-67 is a significant factor in prognosis assessment.^9^ Interestingly, another form of rupture has been ignored, which is GI bleeding caused by mucosal invasion leading to local ischemic necrosis or “being squeezed by the tumor.” Gastrointestinal bleeding is the most common clinical manifestation of SISTs, and the clinicopathological features and prognostic implications of this important manifestation are not well described from population-based perspectives. When GI bleeding is present, uncertainty and debate as to how to evaluate the prognosis continue. The aim of this study is to retrospectively analyze the clinical and pathological characteristics of SIST patients treated at the Zhangzhou Municipal Hospital and to investigate the factors affecting SIST GI bleeding and whether GI bleeding can be used as a predictor of GIST prognosis.

## Materials and Methods

### Patients

A retrospective analysis that included 200 patients diagnosed with SISTs at Zhangzhou Municipal Hospital Affiliated to Fujian Medical University was conducted between January 2013 and December 2022. The inclusion criteria were (1) absence of recurrence or metastasis; (2) absence of other diseases causing gastrointestinal bleeding; (3) no preoperative treatments, including radiotherapy, chemotherapy, imatinib, or other therapies; (4) no other tumors in different locations; (5) complete clinical and pathological data along with regular follow-ups. The criteria for gastrointestinal bleeding were as follows: (1) clinically confirmed bleeding, such as hematemesis or melena; (2) active or recent bleeding identified through endoscopy; and (3) positive fecal occult blood and a decrease in hemoglobin levels, excluding other causes of anemia. Meeting any of these criteria defined the presence of gastrointestinal bleeding. Ultimately, 152 patients were included in this study: 47 with gastrointestinal bleeding and 105 without. This study received approval from Zhangzhou Municipal Hospital Affiliated to Fujian Medical University hospital’s Ethics Committee (202409112157000354680, September 11, 2024). The requirement for obtaining informed consent from patients was waived because the data were stored pseudonymized from the Institutional Ethics Committee of Zhangzhou Municipal Hospital Affiliated to Fujian Medical University. The data utilized in this study, including laboratory tests, pathological examinations, and basic clinical information, were derived from previously hospitalized patients in the institution. All data have been de-identified through the removal of personal identifiers (such as names, identification numbers, and contact details) and are non-traceable to individual patients. Given the retrospective nature of the analysis and the impossibility of re-identifying participants, the study obtained exemption from informed consent from the institutional ethics committee for secondary use of anonymized medical records

### Clinical Data and Follow-Up

This study evaluated clinical, pathological, and histological variables in 152 patients with surgically resected SISTs. The variables included age, gender, tumor location, gastrointestinal hemorrhage, tumor size, mitotic count per 50 high-power fields (HPFs), tumor necrosis, tumor rupture, CD34 positivity, CD117 positivity, Ki-67 (nuclear proliferation index), KIT gene mutation status, and the duration of imatinib treatment. Patients were divided into 2 groups based on whether they experienced gastrointestinal hemorrhage: the GI bleeding group and the non-GI bleeding group.

Follow-up Protocol Summary: For low-risk patients, annual monitoring (combining telephone consultations, WeChat-based imaging submissions, and outpatient visits) was maintained for 5 years. Intermediate/high-risk patients underwent quarterly assessments for the first 3 years, followed by semiannual evaluations until year 5, transitioning to annual follow-up thereafter via outpatient/inpatient visits. For patients with recurrent or metastatic disease, follow-up assessments were conducted quarterly through outpatient clinic visits or hospitalization. Follow-up time was defined as the duration from the date of surgical intervention to the date of the latest follow-up, recurrence, or metastasis. The last follow-up date was May 01, 2023.

### Risk Stratification

Using the 2008 modified NIH risk stratification criteria, all cases were classified into 4 risk groups—very low, low, intermediate, and high—according to factors such as tumor size, location, mitotic count, and rupture.

### Statistical Analysis

All data were analyzed using SPSS 20.0 (IBM SPSS Corp.; Armonk, NY, USA). Categorical variables were compared using a chi-square test, while continuous variables were analyzed with either a Student’s *t*-test or analysis of variance. The Kaplan–Meier method was used to calculate the RFS rate. A log-rank test was used to compare survival curves between the 2 groups in the univariate analysis, with variables showing *P* < .05 included in the multivariate model. Multivariate survival analysis was performed using the Cox proportional hazards model with a stepwise regression approach (forward selection, Entry = 0.05, Removal = 0.1) to identify independent risk factors for RFS. The CI was set between 5% and 95%, with *P* < .05 considered statistically significant.

## Results

### Clinical and Pathological Characteristics

A total of 152 SIST patients were included in this study. The baseline characteristics are shown in Table 1. Among the 152 patients, there were 75 males and 77 females, with a male-to-female ratio of approximately 1:1. Patients’ ages ranged from 28 to 92 years, with a mean age of 57.2 years (±11.4 years) and a median age of 49 years. The mean tumor diameter was 8.98 cm (±3.69 cm); GI bleeding occurred in 47 (30.9%) patients; mitotic count was ≤5/50 HPF in 94 patients (61.8%) and >10/50 HPF in 29 patients (19.1%); 15 (9.9%) patients experienced tumor rupture; 35 (23.0%) patients had tumor necrosis; 46 (30.3%) patients had a Ki-67 index ≤5%; 130 (85.5%) patients were CD34-positive; and 132 (86.8%) patients were CD117-positive. Among 51 cases that underwent genetic testing, *KIT* exon 11 mutations accounted for the highest proportion (41 cases, 80.4%), while *KIT* exon 9 and exon 17 mutations were observed in only 9 cases (17.6%) and 1 case (2.0%), respectively. Analysis of the duration of imatinib treatment revealed that 58 patients (64.4%) received treatment for <3 years, 23 patients (25.6%) for 3-5 years, and 9 patients (10.0%) for over 5 years. Based on the modified NIH criteria, SIST patients were stratified into risk categories as follows: very low risk (6 cases, 4.0%), low risk (44 cases, 28.9%), intermediate risk (19 cases, 12.5%), and high risk (83 cases, 54.6%). No significant differences were found between the GI bleeding group and the non-bleeding group in age, gender, tumor size, nuclear division count, tumor necrosis, CD34 positivity, CD117 positivity, KIT gene mutation status, or duration of imatinib treatment (*P* > .05). However, significant differences were observed in tumor rupture and Ki-67 percentage (*P* < .05) (Table 1).

### Relapse-Free Survival Analysis

Among the 152 SIST patients, 31 cases developed tumor recurrence or metastasis during follow-up, with 16 cases in the GI bleeding group and 15 cases in the non-bleeding group. Recurrence or metastasis occurred between 3 and 69 months post-surgery, with a median time of 35 months. Univariate survival analysis identified several factors affecting RFS in SIST patients, such as nuclear division count (*P* < .001), tumor rupture (*P* < .001), Ki-67 abundance (*P* < .001), and bleeding (*P* = .016). In contrast, other factors such as age, size, tumor necrosis, CD35 positivity, and CD117 positivity showed no association with RFS (Table 2). The 1-, 3-, and 5-year RFSs for the 47 GI bleeding patients were 86.5%, 73.6%, and 59.8%, respectively. For the non-bleeding group, the rates were 98.0%, 92.7%, and 81.3%, respectively (Table 2).

**Figure 1.**
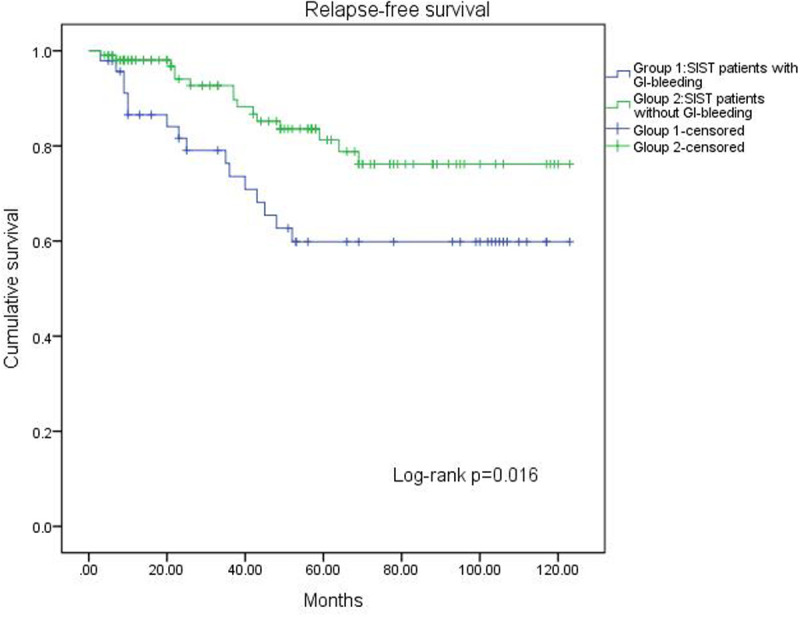
Kaplan–Meier estimates of the RFS of 152 patients in the GI bleeding group and the non-GI bleeding group.

**Figure 2.**
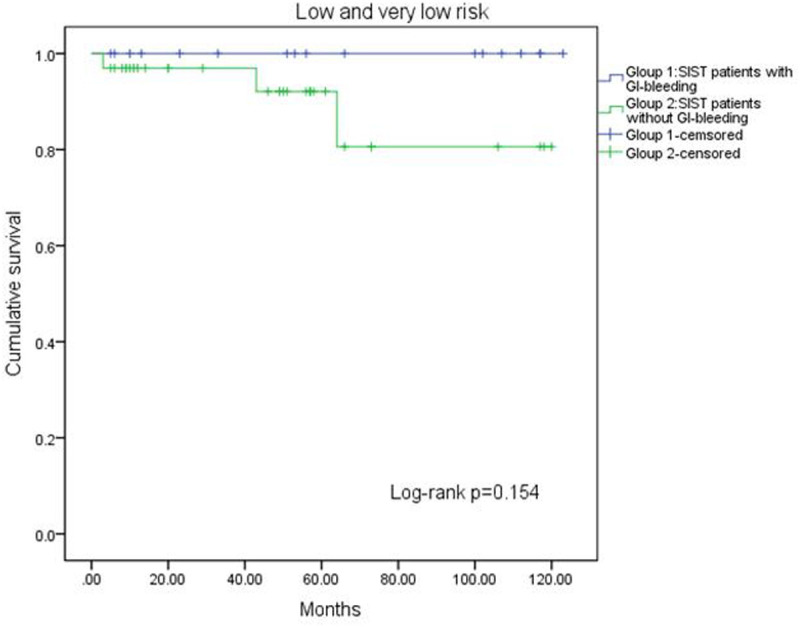
Kaplan–Meier curve analysis showed no difference in relapse-free survival between patients with GI bleeding and non-GI bleeding in the very low-risk group and low-risk group.

**Figure 3.**
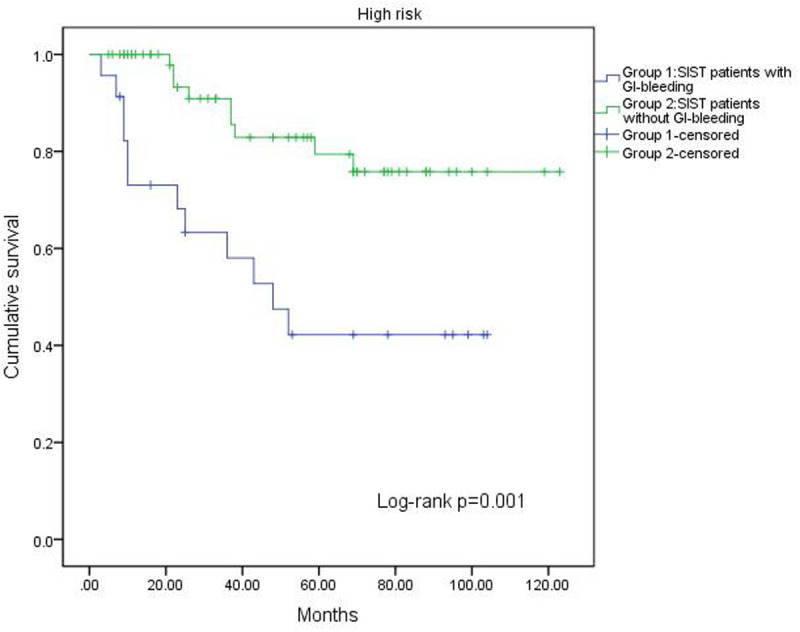
Kaplan–Meier curve analysis showed patients without GI bleeding had longer RFS in high-risk group.

**Figure 4.**
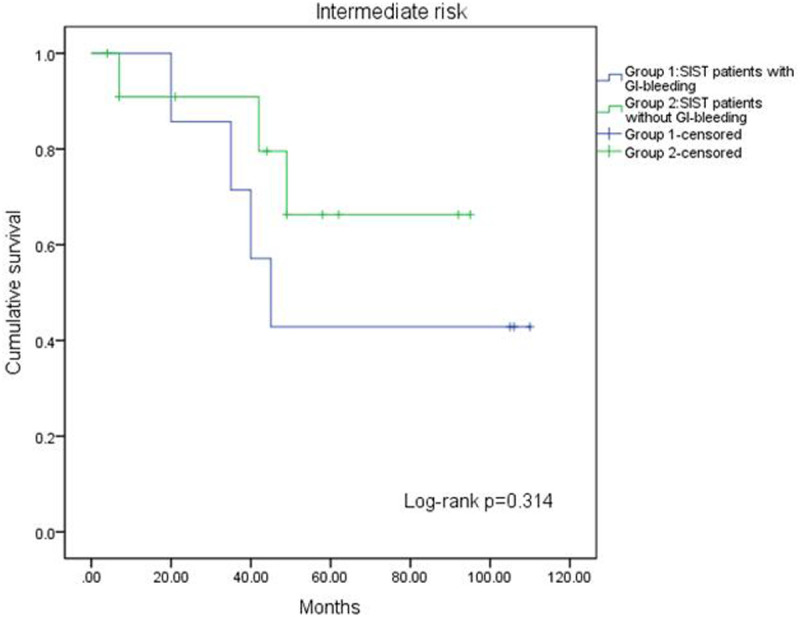
Kaplan–Meier curve analysis showed the effect of GI bleeding on RFS in the intermediate-risk group was uncertain.

A log-rank test confirmed the prognostic factors, revealing that patients without GI bleeding had a longer RFS compared to those with bleeding (*P* = .016, Figure 1). Additionally, the impact of GI bleeding on RFS varied across risk groups. In the very low-risk and low-risk groups, GI bleeding had no effect on RFS (*P* = .154, Figure 2). In the high-risk group, patients without GI bleeding experienced longer RFS (*P* = .001, Figure 3). The effect of GI bleeding on RFS in the intermediate-risk group is unclear, but the data suggest that it may reduce RFS for SIST patients (*P* = .314, Figure 4). Cox regression analysis identified 4 independent predictors of RFS with statistical significance: mitotic count (hazard ratio (HR) = 1.780 (95% CI 1.091-2.904), *P*) = .021, tumor rupture (HR = 2.462 (95% CI 1.081-5.68), *P* = .032], Ki-67 [HR = 2.585 (95% CI 1.085-6.162), *P* = .032), and GI bleeding (HR = 2.114 (95% CI 1.034-4.322), *P* = .040) (Table 3).

## Discussion

Gastrointestinal stromal tumors usually arise from gastrointestinal mesenchymal tissue, primarily located in the stomach and small intestine.^1^ Recent studies indicate that SISTs, a subset of GISTs, are increasingly common and exhibit more aggressive invasive behavior than previously understood.^10^ Prior research has primarily concentrated on gastrointestinal stromal tumors across various locations. However, there have been limited clinical and pathological studies on SISTs, often with small sample sizes. In this retrospective study focusing on SIST patients with gastrointestinal bleeding, it was found that gastrointestinal bleeding is an adverse prognostic factor, particularly pronounced in high-risk patients.

Small intestinal stromal tumors primarily affect older adults, with only a low incidence of 4.6% (7 out of 152 cases in this study) in individuals under 35 years of age. Some studies suggest a male predominance;^11^ however, most literature indicates no significant gender-related differences.^12^^,^^13^ This finding is consistent with the current study’s results, which show comparable male-to-female ratios. The mean age of onset in this cohort was 57 years, which is consistent with the previously reported range of 50 to 57 years.^14^

Small intestinal stromal tumors often have subtle and nonspecific clinical manifestations, making them prone to underdiagnosis, especially in the early stages when the tumors are smaller. Gastrointestinal bleeding represents the most prominent clinical symptom, with literature reporting an incidence ranging from 42% to 66.3%, primarily presenting as recurrent melena or hematochezia.^12^^,^^15^ Currently, there is a growing body of research on gastrointestinal bleeding, with approximately 13.3% of patients experiencing life-threatening hemorrhagic shock, where hemoglobin levels may drop as low as 35 g/L.^12,16^ This study observed a gastrointestinal bleeding incidence of 30.9% (47/152), slightly lower than previous data, suggesting potential heterogeneity. A multivariate survival analysis of 526 patients showed that GIST patients with gastrointestinal bleeding had a better prognosis.^17^ However, this study reached the opposite conclusion—gastrointestinal bleeding was an independent adverse prognostic factor. This discrepancy may stem from the significantly higher tumor rupture rate (15.2% vs. 3.8%, *P* = .048) and elevated Ki-67 expression (68.1% vs. 51.4%, *P* = .032) in the bleeding group, suggesting that hemorrhage may reflect tumor aggressiveness—either vascular invasion due to mucosal infiltration or spontaneous rupture. Combined with high Ki-67 proliferative activity, these factors collectively contribute to poor prognosis in bleeding-associated GIST, providing critical pathological insights for clinical risk stratification.

Studies indicate that tumor location, size, rupture, and mitotic count are key independent prognostic factors for GIST patients.^18^ The modified NIH criteria utilize these indicators to predict prognosis and guide treatment. However, these criteria exclude GI bleeding, an unfavorable prognostic factor for GISTs. Some scholars recommend incorporating GI bleeding into the GIST risk stratification system.^19^^,^^20^ Hølmebakk et al^21^ argue that gastrointestinal bleeding represents a type of tumor rupture, resulting from local mucosal ischemic necrosis or from tumor-induced compression of the gastrointestinal tract. Some researchers suggest that tumor rupture corresponds to R1 resection,^22^^,^^23^ a term that can be ambiguous based on intraoperative judgment. This includes various scenarios, from piecemeal removal of a spontaneously ruptured mass to resection of tumors with microscopic involvement at the margins.^21^ It is not surprising that the reported incidence of rupture in GISTs varies widely, from 2% to 22%.^24^^,25^ Tumor rupture can independently predict the prognosis of GIST patients,^21^ but whether GI bleeding indicates a form of tumor rupture and thus increases the risk of recurrence or metastasis remains unknown. In this study, mitotic count, tumor rupture, Ki-67, and GI bleeding were independent predictors of prognosis in SIST patients. The RFS of SIST patients with GI bleeding was significantly shorter than that of non-GI bleeding patients, along with lower 1-, 3-, and 5-year RFS compared to the non-GI bleeding group. The survival analysis across different risk groups revealed distinct prognostic impacts of gastrointestinal bleeding. In very low-risk and low-risk groups, bleeding showed no effect on RFS, suggesting these cases likely resulted from mucosal injury rather than tumor progression. However, in high-risk patients, gastrointestinal bleeding significantly worsened RFS, potentially reflecting this subgroup’s more aggressive tumor biology, including higher mitotic counts and increased tumor vascularity. While the intermediate-risk group’s results did not reach statistical significance, the observed trend warrants attention and may relate to the limited sample size. These findings emphasize that gastrointestinal bleeding should not be equated with tumor rupture in clinical practice, though potential overlap may exist. Risk-stratified interpretation and management of bleeding symptoms in SIST patients are therefore recommended.

Besides the above indicators, other clinical factors have been investigated. Recent studies have shown that Ki-67 plays a crucial role in tumor progression.^9^ Ki-67 is a nuclear antigen that indicates cell division and proliferation. High levels of Ki-67 are associated with malignant tumor differentiation, invasion, metastasis, and poor prognosis. As a reliable prognostic marker, it has been used to evaluate prognosis in various tumors.^26^ In this study, it was also confirmed that Ki-67 is an independent predictor of prognosis in SIST patients. However, its ability to change the NIH risk classification must be confirmed through larger, multicenter prospective studies.

Previous research consistently shows that postoperative adjuvant therapy with imatinib significantly lowers the risk of recurrence and extends survival. The Z9001 trial found that patients who took 400 mg/day of adjuvant imatinib for 1 year had a 12-month recurrence-free survival (RFS) rate of 97.7%, while the placebo group had an RFS rate of 82.3%.^27^ The SSG XVIII/AIO study further confirmed that high-risk patients treated with 3-year adjuvant imatinib therapy had a 5-year overall survival rate of 92%, significantly higher than the 83% observed in the 1-year therapy group.^28^ However, in this study, some patients delayed starting adjuvant imatinib therapy after surgery due to financial constraints and only began treatment after their tumors recurred. These delays may reduce the potential survival benefits of the treatment. The BFR14 study highlighted that interrupting imatinib therapy accelerates tumor progression, while long-term follow-up data indicated that continuous treatment for over 5 years minimizes recurrence risk.^29^ National research indicates that only a small number of patients in the experimental group completed the recommended 3 years of treatment, leading to significantly higher recurrence rates after stopping the therapy.^30^ To reduce bias caused by varying adherence levels, this study excluded the duration of imatinib treatment from the factor analysis. Additionally, because systematic genetic testing was not conducted for all patients, accurate risk stratification and the inclusion of KIT gene mutations in the analysis were not possible.

Although SISTs are the second most common type of gastrointestinal stromal tumor, their location contributes to a higher level of malignancy compared to tumors in other areas of the gastrointestinal tract. Previous studies reported recurrence rates ranging from 32.5% to 84% and tumor-related death rates between 30.8% and 68%. The 1-year relapse-free survival rate was 85.2%, while the 5-year rate was 43.7%.^31^^,^^32^ In this study, the recurrence rate was 20.4%, which is slightly lower than in previous studies. Additionally, improved 1-year and 5-year RFS rates of 94.4% and 74% were found, respectively, compared to earlier findings, while the 10-year RFS was 79.6%, indicating that SISTs often recur within 5 years of surgery, and individual patients may experience recurrence within 10 years of surgery. Therefore, for patients with SISTs, it is important to enhance both short-term and long-term follow-up after surgery.

This single-center retrospective analysis has 3 main limitations. First, the study design may introduce regional selection bias and data completeness constraints. Second, only a subset of patients received standardized imatinib targeted therapy and systematic genetic testing, which might compromise the comprehensiveness of treatment efficacy evaluation. Third, the compliance rate for imaging follow-up (computed tomography/magnetic resonance imaging) was 88.8% (135/152), with non-compliance primarily attributable to financial constraints among rural patients (15 cases) and contraindications due to renal insufficiency (2 cases). Future multicenter prospective studies with expanded sample sizes and standardized therapeutic monitoring frameworks are needed to enhance research quality.

Gastrointestinal bleeding is a common clinical manifestation in patients with SISTs. Patients with GI bleeding are more likely to experience tumor rupture and have a higher Ki-67 value than those with non-GI-hemorrhagic SISTs. Mitotic count, tumor rupture, Ki-67, and GI bleeding are independent prognostic factors for SISTs. Additionally, the recurrence-free survival (RFS) for patients with GI bleeding is shorter than that for those without, particularly in high-risk patients. It is recommended to enhance follow-up care for these patients.

Critically, the absence of adjuvant imatinib therapy may significantly worsen patient prognosis. Although excluded from multivariable analysis due to data heterogeneity, imatinib remains a potent independent prognostic factor. Clinical decisions should strictly adhere to guideline recommendations.

## Figures and Tables

**Table 1.** Comparison Between the Gastrointestinal Bleeding Group and the Non-Gastrointestinal Bleeding Group

Variable	GI Bleeding (n = 47)	Non-GI Bleeding (n = 105)	*F*	*P*
Age (years)	57.53 ± 11.50	57.02 ± 11.45	0.272	.799
Gender (M/F, n)	26/21	49/56	0.972	.324
Tumor size (cm)	5.73±3.90	6.48±4.25	0.279	.301
Mitotic index (/50 HPF)			4.076	.130
≤5	24	70		
5.1-10	10	19		
>10	13	16		
Tumor rupture			3.914	.048
Yes	8	7		
No	39	98		
Tumor necrosis			0.824	.364
Yes	13	22		
No	34	83		
Ki-67			13.949	<.001
≤5%	24	22		
>5%	23	83		
CD34			0.160	.698
Negative	6	16		
Positive	41	89		
CD117			1.286	.257
Negative	4	16		
Positive	43	89		
Risk			0.934	.627
Very low and low	17	33		
Intermediate	7	12		
High	23	60		
*KIT* (n = 51)			2.548	.280
Exon 11 mutation	18	23		
Exon 9 mutation	2	7		
Exon 17 mutation	0	1		
Duration of imatinib therapy (n = 90) (years)			0.360	.853
<3	16	42		
3-5	5	18		
>5	2	7		

F, female; GI, gastrointestinal; HPF, high-power field; M, male.

**Table 2.** Univariate Survival Analysis of RFS Using the Kaplan–Meier Method for 152 Small Intestinal Stromal Tumor Patients

Characteristics	No. of Recurrences or Metastases/Total	Relapse-Free Survival (%)			*P*
		1 Year	3 Years	5 Years	
Age (years)					.609
≤50	8/46	0.929	0.844	0.784	
51-60	7/41	0.971	0.934	0.737	
>60	16/65	0.971	0.934	0.737	
Size (cm)					.128
≤5	11/75	0.957	0.921	0.824	
6-10	12/55	0.963	0.830	0.710	
> 10	8/22	0.859	0.758	0.565	
Mitotic index (/50 HPF)					<.001
≤5	7/94	0.967	0.951	0.915	
5-10	7/29	0.964	0.878	0.687	
>10	17/29	0.862	0.643	0.407	
Tumor rupture					<.001
No	21/137	0.985	0.914	0.789	
Yes	10/15	0.600	0.467	0.389	
Tumor necrosis					.307
No	22/117	0.936	0.868	0.778	
Yes	9/35	0.971	0.841	0.604	
CD34					.300
Negative	4/22	1	0.952	0.857	
Positive	27/130	0.934	0.845	0.713	
CD117					.361
Negative	3/17	1	0.938	0.875	
Positive	28/135	0.936	0.842	0.719	
Ki-67					
≤5%	8/87	0.951	0.937	0.885	<.001
>5%	23/65	0.936	0.763	0.555	
Risk					
Very low and low	3/50	0.980	0.980	0.948	.013
Intermediate	7/19	0.944	0.822	0.557	
High	21/83	0.923	0.811	0.678	
GI bleeding					.016
No	15/105	0.980	0.927	0.813	
Yes	16/47	0.865	0.736	0.598	

GI, gastrointestinal; HPF, high-power field.

**Table 3.** Cox Regression of RFS for the 152 Small Intestinal Stromal Tumor Patients

Factors	HR	95% CI		*P*
Mitotic count (/50 HPF)	1.780	1.091	2.904	.021
Tumor rupture	2.462	1.081	5.608	.032
Ki-67	2.585	1.085	6.162	.032
GI bleeding	2.114	1.034	4.322	.040

GI, gastrointestinal; HPF, high-power field; HR, hazards ratio.

## Data Availability

The data that support the findings of this study are available on request from the corresponding author.
